# Evaluations of effects of sleep surfaces on athletic performance in youth

**DOI:** 10.1038/s41598-020-68795-5

**Published:** 2020-07-16

**Authors:** Takashi Maruyama, Shinichi Sato, Mari Matsumura, Taisuke Ono, Masaki Nishida, Seiji Nishino

**Affiliations:** 1 Stanford Sleep and Circadian Neurobiology Laboratory, Stanford University School of Medicine, Stanford, CA 94304 USA; 2 Department of Physiology, School of Medicine, University of Occupational and Environmental Health, Kitakyushu, Fukuoka 807-8555 Japan; 3 Department of Neuropsychiatry, Akita University Graduate School of Medicine, Akita, Akita 010-0825 Japan; 4 Faculty of Sports Science, Waseda University, Tokorozawa, Saitama 359-1192 Japan

**Keywords:** Physiology, Neuronal physiology, Sleep disorders

## Abstract

We recently demonstrated that sleeping on high rebound [HR] mattress toppers induced a continuous and more rapid decline in core body temperature compared to low rebound [LR] mattress toppers during the initial phase of nocturnal sleep in young healthy volunteers. HR toppers are characterized by their supportive feel and high breathability whereas LR toppers are pressure-absorbing. In the current study, we evaluated effects of HR mattress toppers on objectively-(actigraphy) and subjectively-(questionnaires) evaluated sleep, vigilance (psychomotor vigilance test), and athletic performance (40-m sprint time, long jump distance, and star drill time) in youth male athletes age 10–19, in two sessions: fifty-one subjects in 2013 (study I) and 23 subjects in 2014 (study II). Sleeping on HR mattress toppers for four to six weeks improved some athletic performance measures compared to sleeping on LR or sleeping directly on spring mattresses without a topper. Statistically significant improvements in 40-m sprint time in study I (compared to LR) and in star drill time in study II (no topper) were observed. No changes in sleep and psychomotor vigilance were observed. These results suggest selecting optimal sleep surfaces may contribute to the maximization of athletic performances, and further studies are warranted.

## Introduction

Sleep has been known to be associated with athletic performance. Sleep deprivation decreases levels of physical performances in a variety of exercises including weight-lifting and endurance exercises such as prolonged treadmill walking^1,2^. More recently, sufficient and restorative sleep has been revealed to be essential to maximize athletic performance for top athletes. Extending sleep hours improved athletic performance in collegiate men’s varsity basketball team members according to a study by Mah et al.^3^. In this study, the athletes first maintained their normal nighttime sleep for 2 to 4 weeks, then maximized their nighttime sleep for the next 5 to 7 weeks. With this extended sleep time, athletic performance improved gradually over the weeks^3^. The athletes ran faster, their shooting accuracy improved by 9%, and their fatigue levels decreased by the end of the study^3^. Subjects also reported improved performances during competitive basketball games^3^.

Subsequent studies have indicated that inadequate sleep negatively affects athletic performance. In semi-professional tennis players, sleep restriction was shown to impair serving accuracy^4^. Restricting sleep of these subject to 5 h (33% reduction) on the night before testing, reduced serving accuracy by approximately 30%. Eighty mg of caffeine intake, which induces a refreshing feeling and counteracts sleepiness, only improved serving performance non-significantly and by a small increment^4^. In a separate study, serving accuracy improved with one week of sleep extension by approximately 1.7 h in collegiate varsity athletes^5^. These results indicate that restoration during sleep may be a significant factor in improving athletic performance.

As for injuries, chronic sleep deprivation increased the risk for athletic injuries in adolescent athletes^6^. Athletes who slept on average < 8 h per night were 1.7 times more likely to have had an injury compared to athletes who slept for ≥ 8 h^6^. These studies reiterate and suggest that adequate sleep is essential for athlete success.

Consequently, in the International Olympic Committee’s recent consensus statement, the importance of sleep in athletics was emphasized^7,8^. The National Collegiate Athletic Association also released a consensus statement that sleep is especially important for young athletes, and insufficient sleep affects health, behavior, attention, learning, and athletic performances^9^.

At the same time, sleep is fragile and can easily be affected by intrinsic factors and by the physical environment. Sleep environment, including factors such as noise, temperature, and light, affects the quality of sleep and daytime performance, and sleep quality can be increased by optimizing these sleep environments^10,11^. A type of sleep environment that is easily manipulatable is sleep surfaces. Mattresses with various types of materials are commercially available and have fairly different characteristics. The limited amount of research present on the effects of sleep surfaces on sleep have shown that sleep surfaces affect sleep in various ways^12–14^.

In fact, we have recently demonstrated that high rebound (HR) mattress toppers induce a continuous and more rapid decline in core body temperature during the initial phase of nocturnal sleep compared to low rebound (LR) mattress toppers in young healthy males^13^. We refer to a type of polyethylene resin fiber-based mattress that has a firm, supportive feel as HR and memory foam mattresses as LR. Polysomnographic evaluations (PSG) showed that an increase in the amount of deep sleep and a predominance of parasympathetic nerve activity occurs in association with the changes in core body temperature^13^. These results suggest that effective heat loss occurs while subjects sleep on HR mattress toppers, and this heat loss may facilitate the occurrence of deeper sleep. The study also demonstrated that rolling over on HR mattress topper requires much less electromyography (EMG) activity than on LR^13^. These findings suggest that HR mattress toppers may facilitate restoration during nocturnal sleep and in turn, may improve daytime performance.

Despite information on how sleep surfaces affect sleep, evaluation of their effects on daytime performances have been limited, and to our knowledge, there are no research regarding the effects of sleep surfaces on athletic performance. In the current study, we evaluated the effects of sleep surfaces on sleep and athletic performance in youth athletes.

## Methods

### Ethics statement

The research protocol including all research designs and evaluations were approved by the IRB of Stanford University (#26470) and by a commercial IRB (Institutional Review Board Services, 372 Hollandview Trail, Suite 300, Aurora, Ontario, Canada) for IMGA. All aspects of the study were performed in accordance to the relevant guidelines and regulations. Informed consent was obtained for all participants except for when participants were minors, in which case legal guardians provided consent.

### Subjects and setting

The study was conducted over two consecutive school years (2013–2015) at the IMG Academy (IMGA) in Bradenton, Florida. IMGA is a private athletic training institute that includes a boarding school for student-athletes who are 6th to 12th graders offering programs including tennis, golf, baseball, basketball, lacrosse, soccer, football, and athletic and personal development^15^. These student-athletes typically train every weekday, and frequently participate in games during the weekends either off-site or on campus.

The study was conducted in two phases, in 2013–2014 in 51 subjects (study I) and in 2014–2015 (study II) in 23 subjects. Student-athletes were recruited from the tennis, baseball, basketball, lacrosse, and soccer programs and were between the age 10–19 at the time of recruitment. Subjects were recruited through flyers posted on the IMGA campus and voluntary information sessions in which the design and purpose of the study, to evaluate if types of mattress toppers affect sleep, vigilance, subjective sleep quality, or athletic performance, was explained. Subjects who were willing to participate in the study and have provided signed informed consent were then screened with the Pittsburg Sleep Quality Index Score [PSQI]^16^, a questionnaire that assesses sleep quality and disturbances. Participants were also asked if they have allergic rhinitis. Fifty one subjects without allergic rhinitis with a mean PSQI score 4.1(± 2.1 [SD], range 1–9) were enrolled for study I, and 23 students without allergic rhinitis with a mean PSQI score 4.5 (± 2.3 [SD], range 3–9) were enrolled for study II (Supplemental Fig. 1 & 2, top). The demographic data of the participants from both study I and II are presented in Table 1.

**Table 1:** Demographic and sleep questionnaire data for the participants.

	Age (years)	Height (cm)	Weight (kg)	PSQI score
Number	Mean ± SD	Mean ± SD	Mean ± SD	Mean ± SD
**Study I**				
51	15.7 ± 3.1	166.3 ± 33.9	69.8 ± 20.5	4.1 ± 2.1
**Study II**				
23	15.1 ± 3.8	169.5 ± 15.8	65.0 ± 18.0	4.5 ± 2.3

### Study design

#### Study I

Fifty-one healthy young male athletes enrolled in 2013–2014. A randomized, cross over design was used to evaluate the effects of HR and LR toppers, both placed on top of the regular beds equipped in the residency halls at IMGA over an 8-week period (Supplemental Fig. 1, top). For HR, a polyethylene resin-fiber-based mattress topper (AIRWEAVE toppers, airweave inc., Tokyo, Japan) and for LR, an urethane-based memory foam mattress topper (Topper Deluxe 3.5, TEMPUR-SEALY Japan Ltd., Kobe, Japan) was used. Half of the athletes slept on HR for the first 4 weeks and then switched to sleeping on LR for another 4 weeks, while the other half first slept on LR for 4 weeks and then on HR for 4 weeks (Supplemental Fig. 1, top). Staff were blind to the treatments. Students were aware of the types of the interventions, but not the hypothesis of the study, to reduce bias.

The subjective sleep quality and performance questionnaires, PVT, and athletic performance tests were administered twice a week in the morning before or immediately following breakfast throughout the study period of 8 weeks. The tests were administered in the order of questionnaires, PVT, and then athletic performance tests. Sleep habits and quantity were also evaluated using an actigraphy device during week 2, 4, 6, and 8.

#### Study II

To replicate the results, an additional study was conducted the following year, in 2014 (Supplemental Fig. 2). Due to school schedule changes, equipment and device availability, and to reduce the expenses, the study design for 2014 was modified and focused on evaluating the effects of HR mattress topper on athletic performance.

Twenty-three healthy young male athletes were enrolled in 2014. A randomized-control, cross-over design was used to evaluate the effects of sleeping with or without HR-toppers placed on regular beds equipped at the residence halls of IMGA. Half of the athletes slept with HR for the first 6 weeks and switched to sleeping without toppers for the next 6 weeks, while the other half of the athletes first slept without toppers for 6 weeks and then with HR for 6 weeks. Staff were blind to the treatments. Students were aware of the types of the interventions, but not the hypothesis of the study, to reduce bias.

Athletic performance tests, subjective sleep quality and performance questionnaires, and PVT were administered twice a week during last 2 weeks of each 6 weeks topper session (week 5, 6, 11, and 12). The tests were administered in the order of questionnaires, PVT, and then athletic performance tests and were administered immediately before or after breakfast.

### Athletic performance

To evaluate athletic performance, long jump distance [LJ], 40-m sprint time [SP], and star drill time [SD] were collected for both study I and II (Supplemental Fig. 1 and 2). The athletic measurements were selected in consultation with the IMGA coaches as measures that best reflect the performance level in the participants’ sports: tennis, baseball, basketball, lacrosse, and soccer.

For long jump, athletes stood behind a line marked on the ground with their feet slightly apart. The subjects were instructed to jump as far as possible and to land on both feet without falling backwards. The distance between the take-off line and the nearest point of contact on the landing (backside of the heels) was measured. The better of two attempts were recorded.

For the 40-m sprint, students performed a single maximum effort sprint over 40 m, and the times were recorded. Students started from a stationary position with one foot in front of the other and one foot on the starting line. The time was recorded by a person at the finish line with a stopwatch as the students crossed the finish line.

For the star drill, cones were laid out with four cones placed in a square shape and one cone in the middle; the outer cones were each placed 3 m from the center cone (Supplemental Fig. 1, bottom). Athletes started with their left hand on the middle cone, facing forward, then ran to the right to cone 2. They then turned back and ran to the center cone, out to cone 3, back to the center, out to cone 4, back to the center and finished the drill by running through the finish line at cone 5. The athletes were required to touch the cone with their hand at each turn. The timer was started when the hand came off the center cone and was stopped when the chest passed through the line of the final cone.

### Subjective sleep quality and performance questionnaire

Subjects were asked to provide various self-ratings in questionnaires. For both study I and II, a self-rating of how the subjects’ felt their athletic performance level was at practice (SSRP) and during games (SSRG) were collected in a 10 point Likert scale (1 = worst, 10 = best). On the same questionnaire, subjects self-evaluated their sleep quality and overall performance level using visual analogue scales for sleep [VAS-S] (very bad to very good) and performance level [VAS-P] (very bad to very good)^17^. For study II, a visual analogue scale for mood [VAS-M] (very bad to very good) was also added. Lastly, the epworth sleepiness scale (ESS)^18^ was administered to track daytime sleepiness of the participants in both study I and II. Hard copy forms of questionnaire were handed out and filled out by the participants on every evaluation day, before the PVT and the athletic performance testing.

### PVT

To objectively measure alertness and vigilance levels, a standardized psychomotor vigilance test (PVT)^19^ was used in both study I and II. PVT is a sustained-attention, reaction-timed task that measures the speed with which subjects respond to a visual stimulus^19^. Increased sleep debt or deficit correlates with deterioration in alertness, slower problem-solving, declined psycho-motor skills, and increased rate of false responding^19^. A PVT software developed by Walter Reed Army Institute of Research for Palm-OS-based personal data assistants (PDAs)^20^ was installed and used on Sony CLIE, PEG-SJ33/U (Sony, Tokyo, Japan), a PDA device. This version takes five minutes to complete and was conducted immediately after the questionnaires and before the athletic performance testing.

### Actigraphy

To objectively evaluate the subjects’ sleep habits and sleep quantity during the study, subjects wore ActiGraph GT3X + , an actigraphy device (ActiGraph, Pensacola, FL) on their wrists during week 2, 4, 6, and 8 of study I. Subjects were asked to wear the device on their non-dominant arm for 24 h a day for those weeks starting on Mondays, and were instructed to take the devices off during their practice/game times and when they showered^21^. The actigraphy data were scored by one of the authors (S. S.) blinded to the subject and session information. The scorer followed the SBSM actigraphy guidelines^22^ and manually adjusted the bed interval on the automatic scoring data using the Cole–Kripke algorithm.

### Statistical analysis

Since effects on athletic performance are likely to appear several days after installing a new mattress topper, the latter 2 weeks of each topper intervention was compared.

For study I, significances of the effects (between HR and LR) on the mean scores between the second 2-weeks (week 3 and 4) were analyzed with the Wilcoxon rank sum test using JMP (SAS Institute, Cary, North Carolina). In addition, in order to evaluate the time course changes of each testing parameter, repeated measures ANOVA was applied to the first 2-weeks (week 1 and 2) and second 2-weeks (week 3 and 4) with topper type as the grouping factor.

For study II, significances of the effects on the mean scores (week 5 and 6) between HR and no topper were analyzed with the Wilcoxon rank sum test. In order to evaluate the time course changes of each testing parameter, repeated measures ANOVA was applied to each week’s data with topper type as the grouping factor.

## Results

### Study I (2013)

Among the 51 participants, 47 subjects participated in both sessions, and paired data were obtained from 28 to 39 subjects, depending on the measure. The combined 3rd and 4th week data of each topper sessions for subjective sleep evaluations (questionnaires), psychomotor vigilance tests (PVT), objective sleep evaluations (actigraphy) and objective and subjective athletic performance (self-ratings of athletic performance level, long jump, 40-m sprint, and star drill) (see supplemental Fig. 1) are presented in Table 2.

**Table 2:** Sleep and athletic performance evaluations in Study I.

	Number	HR-topper	LR-topper	p-value
		Mean ± SEM	Mean ± SEM	
**Subjective evaluation**				
ESS	34	8.41 ± 0.86	9.02 ± 0.80	0.66
VAS-S	38	2.18 ± 0.25	1.90 ± 0.24	0.21
VAS-P	39	2.17 ± 0.27	1.90 ± 0.23	0.11
PMOS	39	7.76 ± 1.57	8.45 ± 1.53	0.68
SSRP	39	7.50 ± 0.21	7.86 ± 0.22	0.07
SSRG	39	7.64 ± 0.24	7.83 ± 0.24	0.11
**PVT**				
MeanRT (msec)	39	362.99 ± 25.17	344.42 ± 19.52	0.66
MajorLapses (#/5 min)	39	0.33 ± 0.15	0.28 ± 0.09	0.59
MinorLapses (#/5 min)	39	8.17 ± 1.29	7.96 ± 1.30	0.83
**Actigraph**				
Total Minutes in Bed	28	511.51 ± 42.89	529.11 ± 43.31	0.32
Latency (min)	28	9.39 ± 1.90	9.63 ± 1.56	0.70
Total Sleep Time (min)	28	440.42 ± 45.73	457.71 ± 45.60	0.43
**Athletic performance**				
Long Jump (cm)	31	181.8 ± 4.6	179.8 ± 4.8	0.72
40 M sprint (sec)	31	7.00 ± 0.15	7.28 ± 0.13	0.06
Star Drill (sec)	31	31.82 ± 0.58	32.00 ± 0.75	0.45

There were no significant differences in objective sleep measures and vigilance (actigraphy and PVT) nor subjective sleepiness and sleep quality (ESS and VAS-S) between HR and LR sessions (Table 2). There were also no significant differences in subjective performances levels (VAS-P) nor self-ratings of athletic performance level (SSRP and SSRG) between HR and LR sessions. For athletic performance, we observed an improvement of 0.28 s which was marginally significant (p = 0.06), in the 40 m sprint with HR use ([HR vs. LR] 40-m sprint (n = 31): 7.00 ± 0.15 vs. 7.28 ± 0.13 s) (Table 2). Improvements for long jump and star drill however, did not reach statistical significance ([HR vs. LR] long jump (n = 31): 181.8 ± 4.6 vs. 179.8 ± 4.8 cm p = 0.72, start drill (n = 31): 31.82 ± 0.58 vs. 32.00 ± 0.75 s p = 0.45) (Table 2).

Results combined from the first 2-weeks of sleeping on each topper showed that improvements in long jump, 40-m sprint, and star drill with HR was not statistically significant ([HR vs. LR] long jump (n = 31): 181.2 ± 5.0 vs. 179.8 ± 5.0 cm p = 0.71, 40-m sprint (n = 31): 6.78 ± 0.13 vs. 7.06 ± 0.14 s p = 0.11, star drill (n = 31): 31.74 ± 0.51 vs. 32.09 ± 0.51 s, p = 0.50).

**Figure 1:**
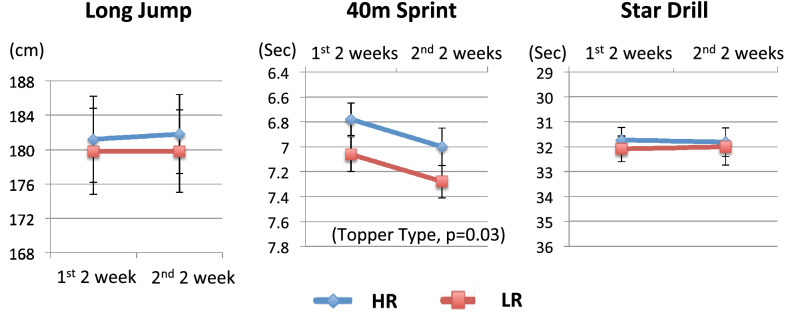
Athletic performance after sleeping with HR-toppers vs. LR in 2013 (Study I). The repeated measures ANOVA for the first 2-weeks (week 1 and 2) and second 2-weeks (week 3 and 4) data revealed that improvement in 40-m sprint by topper was significant (topper: F(1, 60) = 5.19, p = 0.03, time: F(1, 60) = 0.35, p = 0.56, topper x time: F(1, 60) = 0.24 p = 0.62). Improvements for long jump (topper: F(1, 60) = 0.21 p = 0.64, time: F(1, 60) = 0.13, p = 0.72, topper x time: F(1, 60) = 0.003, p = 0.95) and star drill (topper: : F(1, 60) = 0.099 p = 0.75, time: F(1, 60) = 0.014, p = 0.90, topper × time: F(1, 60) = 0.26, p = 0.61) did not reach statistically significant levels. Values in y-axis for 40-m sprint and star drill are displayed in reverse direction, in order to present better performances higher on the y-axis. The values are displayed as mean ± SEM.

Applying the repeated measures ANOVA to the first 2-weeks (week 1 and 2) and second 2-weeks (week 3 and 4) data revealed that improvement in 40-m sprint by topper was statistically significant (topper: p = 0.03, time: p = 0.56, topper x time: p = 0.62) (Fig. 1), but improvements for long jump (topper: p = 0.64, time: p = 0.72, topper x time: p = 0.95) and star drill (topper: p = 0.75, time: p = 0.90, topper x time: p = 0.61) were not statistically significant (Fig. 1). In contrast, no significant changes were seen in subjective self-ratings of athletic performance level (SSRP and SSRG), objective (actigraphy) and subjective (ESS and VAS-S) sleep, and objective psychomotor performance (PVT) between HR and LR sessions by repeated measures ANOVA (type, time, type × time, p > 0.05). Improvements in the 40-m sprint time with HR were observed regardless of the order of the topper use.

### Study II (2014)

Among the 23 participants, paired data were obtained from 21 to 23 subjects, depending on the measure. The mean values of the week 5 and 6 data for subjective sleep evaluations (questionnaires), PVT, and athletic performance (self-ratings of athletic performance level, long jump, 40-m sprint, and star drill) (see also supplemental Fig. 2) are presented in Table 3. There were no significant differences in the subjective self-ratings of athletic performance level (SSRP and SSRG), subjective sleep, performance, and mood (VAS-S, VAS-P, and VAS-M), as well as objective psychomotor performance (PVT) between with or without the use of HR (Table 3).

**Table 3:** Sleep and athletic performance evaluations in Study II.

	Number	HR-topper	Non-topper	p-value
		Mean ± SEM	Mean ± SEM	
**Subjective evaluation**				
ESS	23	9.92 ± 0.89	9.21 ± 0.91	0.23
VASs	23	7.25 ± 0.30	6.89 ± 0.32	0.43
VASp	23	7.07 ± 0.32	6.98 ± 0.29	0.89
VASm	23	7.14 ± 0.30	7.17 ± 0.30	0.62
SSRP	23	7.32 ± 0.26	7.43 ± 0.21	0.84
SSRG	23	7.46 ± 0.31	7.38 ± 0.24	0.34
**PVT**				
MeanRT	23	333.96 ± 27.22	343.27 ± 18.68	0.29
MajorLapses	23	0.22 ± 0.23	0.09 ± 0.07	0.91
MinorLapses	23	6.22 ± 1.21	7.51 ± 1.25	0.07
**Athletic performance**				
Long Jump (cm)	21	168.8 ± 5.0	167.5 ± 5.3	0.71
40 M sprint (sec)	21	7.16 ± 0.15	7.22 ± 0.14	0.14
Star Drill (sec)	21	31.03 ± 0.59	32.83 ± 0.67	0.04

**Figure 2:**
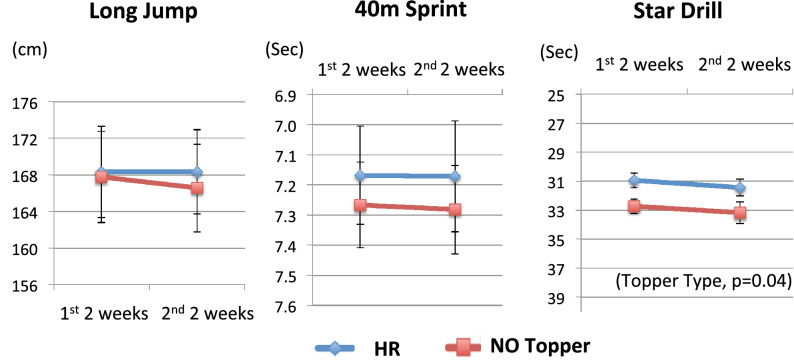
Athletic performance after sleeping with HR-toppers vs. LR in 2014 (Study II). The repeated measures ANOVA for the week 5 and week 6 data revealed improvements in the star drill with HR-topper use (topper: F (1,40), 4.41 p = 0.04, time: F (1,40) = 0.25, p = 0.62, topper x time: F(1, 40) = 0.0009, p = 0.98). Improvements in the long jump (topper: F (1,40) = 0.028, p = 0.87, time: F(1, 40) = 0.19, p = 0.66, topper x time: F(1,40) = 0.69, p = 0.41) and 40-m sprint (topper: F(1, 40) = 0.14, p = 0.71, time: F(1, 40) = 0.038, p = 0.85, topper x time: F(1, 40) = 0.077, p = 0.78) did not reach significant levels. Values in y-axis for 40-m sprint and star drill are displayed in reverse direction, in order to present better performances higher on the y-axis. The values are displayed as mean ± SEM.

We observed statistically significant improvements in start drill time with HR-topper use ([HR vs. no topper]: 31.03 ± 0.59 vs. 32.83 ± 0.67 s, n = 21, p = 0.04) (Table 3). Improvements in the long jump distance (168.8 ± 5.0 vs. 167.5 ± 5.3, n = 21, p = 0.71) and 40-m sprint time (7.16 ± 0.15 vs. 7.22 ± 0.14 s, n = 21, p = 0.14) with the use of HR, however, were not statistically significant. We also analyzed the week 5 and 6 data with the repeated measures ANOVA by topper type, and found statistically significant improvements in star drill with HR-topper use (topper: p = 0.04, time: p = 0.62, topper x time: p = 0.98), but not in long jump (topper: p = 0.87, time: p = 0.66, topper x time: p = 0.41) nor 40-m sprint (topper: p = 0.71, time: p = 0.85, topper x time: p = 0.78) (Fig. 2).

Similarly, no significant changes were observed in the subjective self-ratings of athletic performance level (SSRP and SSRG), subjective sleep, performance, and mood (VAS-S, VAS-P, and VAS-M), as well as objective psychomotor performance (PVT) between HR and LR sessions by repeated measures ANOVA (type, time, type × time, p > 0.05).

## Discussions

In the current study, we evaluated if sleeping with HR mattress toppers improves sleep and athletic performance in youth male student-athletes since HR mattress toppers induce increase in deep and restorative sleep at the initial phase of nocturnal sleep in healthy adult male subjects^13^.

Since effects on athletic performance are likely to appear several days after installing a new mattress topper, we first analyzed performances in the latter 2-weeks of the 4-week topper sessions in study I. We observed improved performance of marginally significant levels in 40-m sprint during the latter 2-weeks of the 4-week topper sessions (Table 2). Improvements in long jump distance and star drill time did not reach statistical significance. Similar trends were also seen during the first 2-weeks. The repeated measures ANOVA of the results from the first and second two weeks’ data revealed that improvement in 40-m sprint by topper was statistically significant (Fig. 1), suggesting sleeping with HR improves some athletic performance measures in youth athletes.

In order to replicate the results of study I, we conducted an additional study (study II) the next year. In study II, we observed statistically significant improvements in star drill time associated with sleeping on HR mattress toppers, for the mean of data combined from the 5th and 6th week (Table 3). The repeated measures ANOVA for data from the 5th and 6th week revealed that improvements in star drill with HR mattress toppers were statistically significant. Improvements in the long jump and 40-m sprint with HR mattress toppers (Table 3) were neither statistically significant for the combined data from the 5th and 6th week nor for the repeated measures analysis of each week.

Based on these results, we believe that sleeping with HR mattress toppers likely improves some athletic performances measures in youth, although the significant improvements we observed were in different types of performance measures in each study, and results are still preliminary.

These improvements in athletic performances with HR mattress toppers may be due to increases in sleep quantity and improvements in qualities. We however could not detect any improvements in the subjective reporting of sleep, performance, mood, athletic performance, nor objective sleep and psychomotor performance between HR mattress toppers and LR mattress toppers and with vs. without HR mattress toppers.

The HR mattress toppers used in this study contain a large amount of air inside the mattress, increasing breathability, as thin fishing line-like material is woven to construct the basic three-dimensional structure of the mattress^23^. HR mattress toppers have been previously shown to be associated with declines in core body temperature during the initial phase of nocturnal sleep, and less EMG activities of the upper body were observed to roll on HR mattress toppers compared to on LR^13^. In contrast, the sleep improvements were relatively small and were only significant with detailed sleep analysis, such as PSG sleep staging or sleep EEG frequency analysis under strictly-controlled laboratory settings^13^. Subjective feelings of improved sleep were also observed with HR mattress toppers though at statistically non-significant levels^13^. Possibly, we were unable to detect subtle improvements in sleep related parameters associated with mattress toppers as field studies are less controlled and use indirect measures of sleep such as actigraphy.

Considering the larger declines in body temperature and the smaller EMG activities required in the upper body for roll-over, sleeping on HR mattress toppers likely produces more profound restoration during sleep, and this also explains why better performances were observed when the subjects slept with HR mattress toppers.

As far as we know, this is the first study evaluating the effects of different types of matters toppers on sleep and athletic performances. The physique of the subjects, such as body weight or height, have been reported to influence the preferences of the mattress types; heavy and taller subjects tend to prefer harder mattresses^24^. Although there has been no data suggesting that sleeping with preferred types of mattress induce better sleep and performance so far, the role of bedding preference in sleep and performance may be another important aspect to further study in relation to the effects of mattress on sleep and athletic performances.

Athletes often experience poor quality of sleep whether at the elite or collegiate level, and travel is reported as one of the factors associated with poor sleep^25,26^. Sleep quality can decrease when athletes sleep in unfamiliar settings^27^, and especially as top athletes often tour domestically or internationally, they may encounter unfamiliar or uncomfortable sleep surfaces, which may affect their sleep, restoration, and athletic performances. The importance of sleep surfaces has garnered little attention compared to other travel-related factors such as jet-lag or local weather, but may be one of the solutions for sleep problems that arise due to unfamiliar settings.

The current study is still preliminary, and only included male athletes, but suggests the importance of mattress and sleep surface selection in relation to athletic performance. Further studies about bedding conditions and sleep and athletic performances are warranted.

## Supplementary information

Supplementary Information.
