# Supplementary material for: Evaluations of effects of sleep surfaces on athletic performance in youth
Source: Sci Rep. 2020 Jul 16;10:11805. doi: 10.1038/s41598-020-68795-5 (PMC7366624; doi:10.1038/s41598-020-68795-5)
Supplement: Supplementary file 1 — Supplementary Information. [file 41598_2020_68795_MOESM1_ESM.pdf]

Supplementary Information

Evaluations of Effects of Sleep Surfaces on Athletic Performance in Youth

Takashi Maruyama[1, 2]
Shinichi Sato[1, 3]
Mari Matsumura[1]
Taisuke Ono[1]
Masaki Nishida[1, 4]
Seiji Nishino[1]*

1. Stanford Sleep and Circadian Neurobiology Laboratory
Stanford University School of Medicine, Stanford, CA, 94304, USA
2. Department of Physiology, School of Medicine, University of Occupational and Environmental Health, Kitakyushu, Fukuoka, 807-8555, Japan
3. Department of Neuropsychiatry, Akita University Graduate School of Medicine, Akita, Akita, 010-0825, Japan
4. Faculty of Sports Science, Waseda University, Tokorozawa, Saitama, 359-1192, Japan

*Correspondence to [nishino@stanford.edu]

Supplemental Figure 1

IMGA Study Evaluations 2013

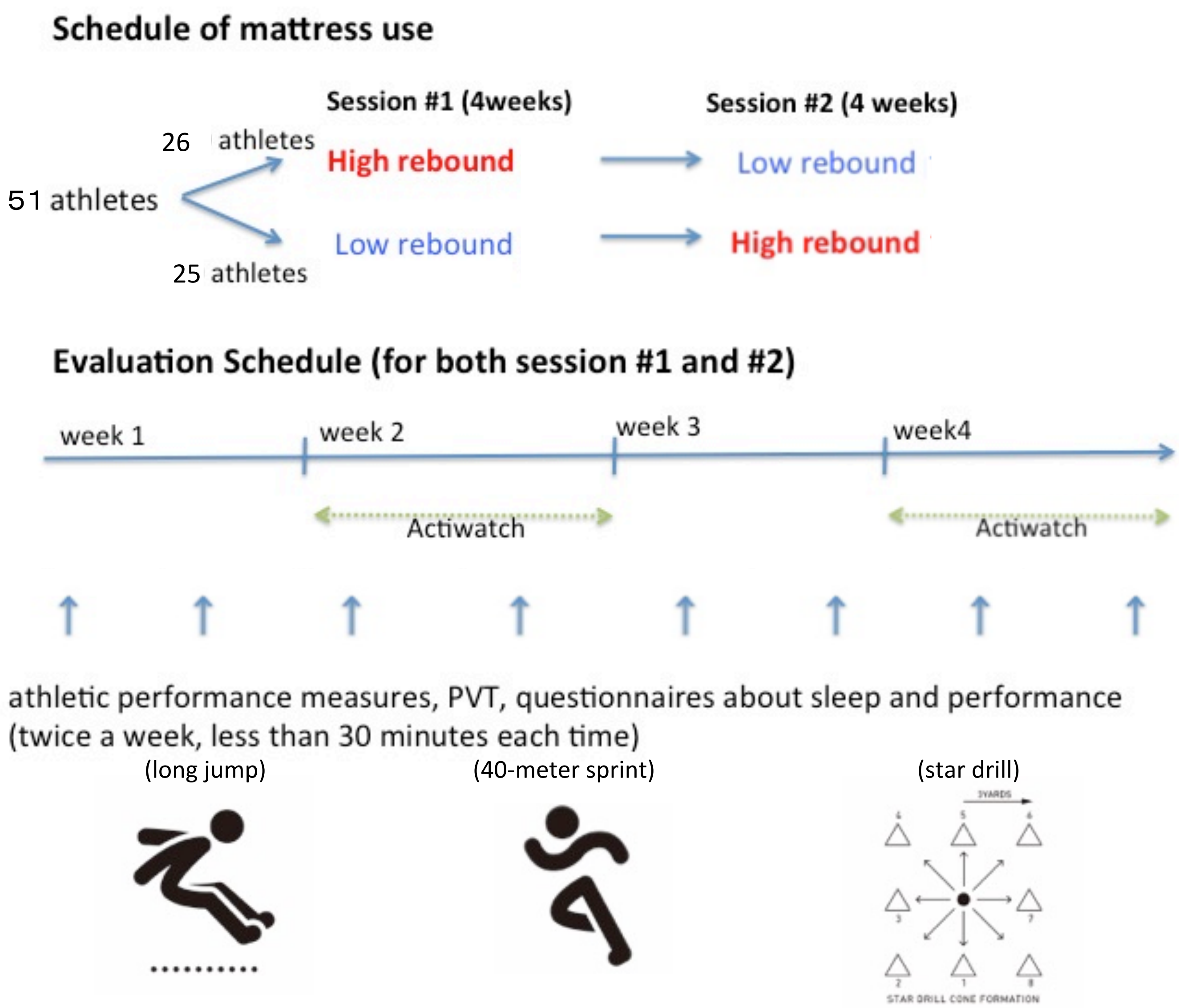

Half of the participants will start by 4 weeks of using high rebound mattress toppers followed by 4 weeks with low rebound mattress toppers, while the order of the sessions was reversed for the other half. Sleep and athletic performance will be evaluated during these 8 weeks.

Supplemental Figure 2

IMGA Study Evaluations 2014

Schedule of mattress use

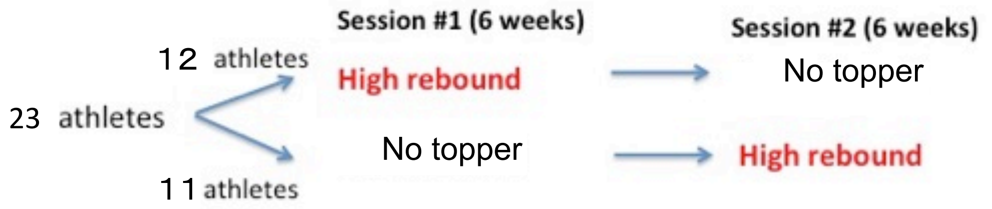

Evaluation Schedule

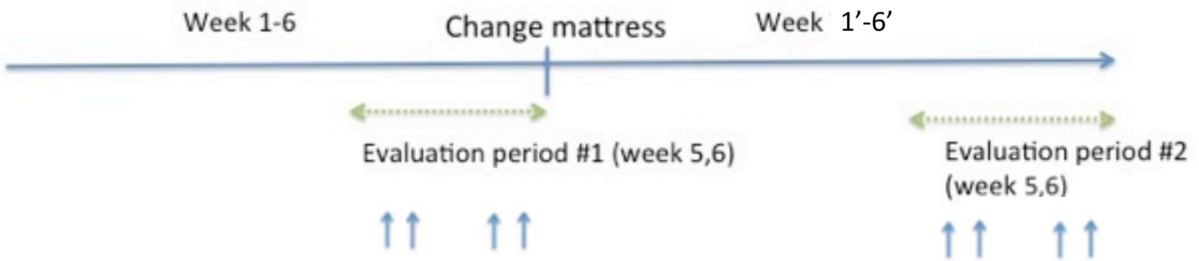

Evaluation period (weeks 5,6): athletic performance measures, PVT, questionnaires about sleep and performance (twice a week)

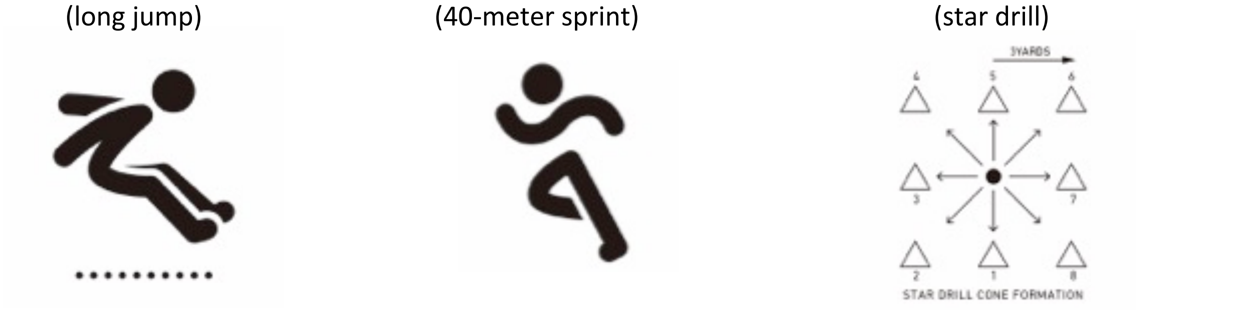

- Half of the participants will start by 6 weeks of using high rebound mattress toppers followed by 6 weeks with no mattress toppers, while the order of the sessions was reversed for the other half. Sleep and athletic performance were evaluated in week 5, 6.
